# Multi‐Modality Imaging in Coronary Anomalies: Focus on Anomalous Aortic Origin of Coronary Arteries

**DOI:** 10.1111/echo.70213

**Published:** 2025-06-06

**Authors:** Guglielmo Capponi, Biagio Castaldi, Domenico Sirico, Elena Reffo, Nadia Assanta, Giuseppe Santoro, Francesco Prati, Alice Pozza, Massimiliano Cantinotti, Giovanni Di Salvo

**Affiliations:** ^1^ Department of Pediatric Cardiology and Congenital Heart Disease National Research Council‐Tuscany Region G. Monasterio Foundation (FTGM) Massa Pisa Italy; ^2^ Pediatric Cardiology Unit, Department of Woman's and Child's Health University‐Hospital of Padova University of Padua Padua Italy

**Keywords:** angiography, coronary arteries anomalies, flow‐chart, imaging, physical activity

## Abstract

Coronary artery anomalies represent a series of congenital heart diseases characterized by the abnormal circulation of coronary arteries. Of particular interest is the anomalous aortic origin of coronary arteries (AAOCA), an underdiagnosed but potentially fatal anomaly, specifically relevant in young adult athletes. During the last decades, knowledge of the pathophysiology of AAOCA‐related ischemic mechanisms has increased, and major risk conditions have been discovered. Unfortunately, a universally shared approach is still lacking, and clinicians often follow the policy of their own centers. Most of the population with AAOCA is asymptomatic or complains of minor symptoms but is among them that there is much more uncertainty about the correct strategy to perform a risk stratification process, and consequently for providing indications on exercise restriction or surgery. This review focuses on the diagnostic approach to coronary anomalies and discusses the diagnostic workflow in these patients. Finally, we perform an in‐depth analysis of novel angiographic invasive functional methods and how they contribute to AAOCA in selected cases.

## Graphical abstract

The study of anomalous coronary arteries involves a multidisciplinary evaluation, primarily focusing on echocardiographic assessment. It then requires confirmation with coronary CT angiography, and in doubtful cases, invasive methods like angiography may be necessary. Through angiography, coronary physiology can be studied using iFR or FFR techniques.

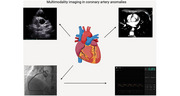

## 1 Introduction

Coronary artery anomalies (CAA) include a series of congenital heart diseases characterized by an abnormal origin, course, or termination of one or more of the three epicardial coronary arteries [1].

The classification proposed by Angelini [1] divides CAA into anomalous origin of the coronaries from the pulmonary artery, for example, the origin of the left main coronary artery from the pulmonary artery (ALCAPA) or the origin of the right coronary artery from the pulmonary artery (ARCAPA); anomalous aortic origin of the coronary arteries (AAOCA), and congenital atresia of the ostia; anomalies of the course, like myocardial bridges, coronary aneurisms, coronary fistulas, and coronary stenoses [1].

The true incidence and clinical relevance of coronary anomalies are still unknown because most of these lesions might remain throughout life, and often, AAOCA might be an incidental diagnosis. On the other hand, some variants have been recognized as a cause of life‐threatening events [2, 3]. In 2000, Basso et al. [4] identified a cohort of 27 young competitive athletes who died during or shortly after strenuous exertion and were found at autopsy to have a congenital aortic origin of a coronary artery from the wrong sinus. In 2009, Maron et al. [5] presented a large registry including 1866 American athletes who had died suddenly or survived a cardiac arrest over 27 years of observations: among the 1049 cardiovascular deaths, the most common causes were hypertrophic cardiomyopathy (30%) and CAA (17%).

The incidence of major anomalies of coronary arteries varies significantly and is highly dependent on the imaging tool used. According to pathological studies, the incidence varies from 0.2% to 2.2%, while in computed tomography (CT) series, CAA has been observed in 0.3%–1.8% of cases. In echocardiography, the rate ranges from 0.19% to 0.86% [6, 7, 8, 9, 10, 11]. From a clinician's point of view, the real challenge is to identify, among CAA, the variants with the higher risk of life‐threatening events.

In the last decade, a growing interest has focused on AAOCA patients. A large amount of data is available on multimodality imaging, proposed diagnostic workup strategies, surgical management, and sports practice. The latest adult congenital heart disease guidelines indicated high‐risk items, such as an intramural course or a slit‐like orifice, acute take‐off, and orifice > 1 cm above the sinutubular junction (STJ), as factors guiding the choice towards a surgical strategy rather than a watchful waiting approach [12]. Unfortunately, the management proposed for adult patients cannot be indiscriminately applied to children.

In this paper, the role of multimodality imaging in CAA patients is discussed. We evaluated the role of echocardiography, CT, cardiac magnetic resonance (CMR), and angiographic invasive functional imaging using the instantaneous wave‐free ratio (iFR)‐fractional flow reserve (FFR) and/or intravascular ultrasound (IVUS) to help the decision‐making process in different settings.

## 2 Echocardiography

Transthoracic echocardiography (TTE) remains the key examination in the diagnostic work‐up of CAA. In children and young adults, TTE allows an optimal visualization of coronary ostia without radiation exposure [13, 14]. The accuracy depends mostly on the patient's age, on the acoustic window quality, and on the expertise of the echocardiographer. In ideal settings (young patients, high‐performance echo machine, and skilled operators), the accuracy in identifying the left coronary artery origin ranges from 98% to 100%, while for the right coronary artery, it varies from 80% to 96% [9, 15, 16, 17, 18]. Furthermore, echocardiography helps with the delineation of the first segment of both vessels in about 80% of cases [16]. Thus, TTE may be considered the first‐line tool to evaluate AAOCA [19, 20]. In the past decade, several studies underlined the possibility of stratifying AAOCA based on echocardiographic findings [18, 19, 20]. Turner et al. [21] showed that echocardiography predicted intramural or extramural course in 92.5% of AAOCA patients. In a more recent study, Lorber et al. [22] introduced a TTE protocol to define a precise evaluation of coronary arteries regarding their origin, course, and relationship with the great arteries. An acute angle of origin and diastolic flow on color Doppler in the proximal course of the AAOCA within the aortic wall correctly defined intramural course in 94% of patients [22].

A high coronary take‐off is a possible CAA feature. In adult patients, the threshold for a high take‐off is 1 cm over the sinotubular junction. In the latest guidelines [12], it was proposed as a major risk factor for sudden cardiac death. From the surgical point of view, a coronary origin above Valsalva sinuses might facilitate surgical unroofing maneuver because it does not require commissural resuspension [22].

In children, theclinical significance of a high take‐off is still debated, because the aortic size changes progressively with somatic growth [9, 12]. Therefore, different parameters have been proposed in children: origin of 120% or more of the depth of sinus of Valsalva or 20% or more of the depth of sinus above the STJ [23, 24]. Cantinotti et al. identified an association between high take‐off and coronary origin in the axial plane. Using the center of the valve as a reference point, they analyzed the angle formed between the coronary artery and the apex of the probe. In the short‐axis view, a right coronary artery emergence angle of < 18.5° predicted high take‐off with very high sensitivity (98.3%) and specificity (93.1%). Conversely, a high left main coronary artery take‐off was associated with an emergence angle of > 119.5°, with a sensitivity of 70.6% and specificity of 82.4% [9].

Stress echo may also provide functional information of AAOCA through exercise or dobutamine‐stress testing in pediatric patients [25, 26, 27]. In a pediatric setting, Kimbal et al. demonstrated that dobutamine exercise test was diagnostic in 84% of patients (due to symptoms and for target heart rate achievement), and in 19% of them, a pattern suggestive of ischemia was induced [28].

In adult patients, or in case of poor transthoracic acoustic window, transesophageal echocardiography may be more appropriate to study coronary arteries in patients at risk of or suspected to have AAOCA [2, 29].

## 3 Cardiac CT

**FIGURE 1:**
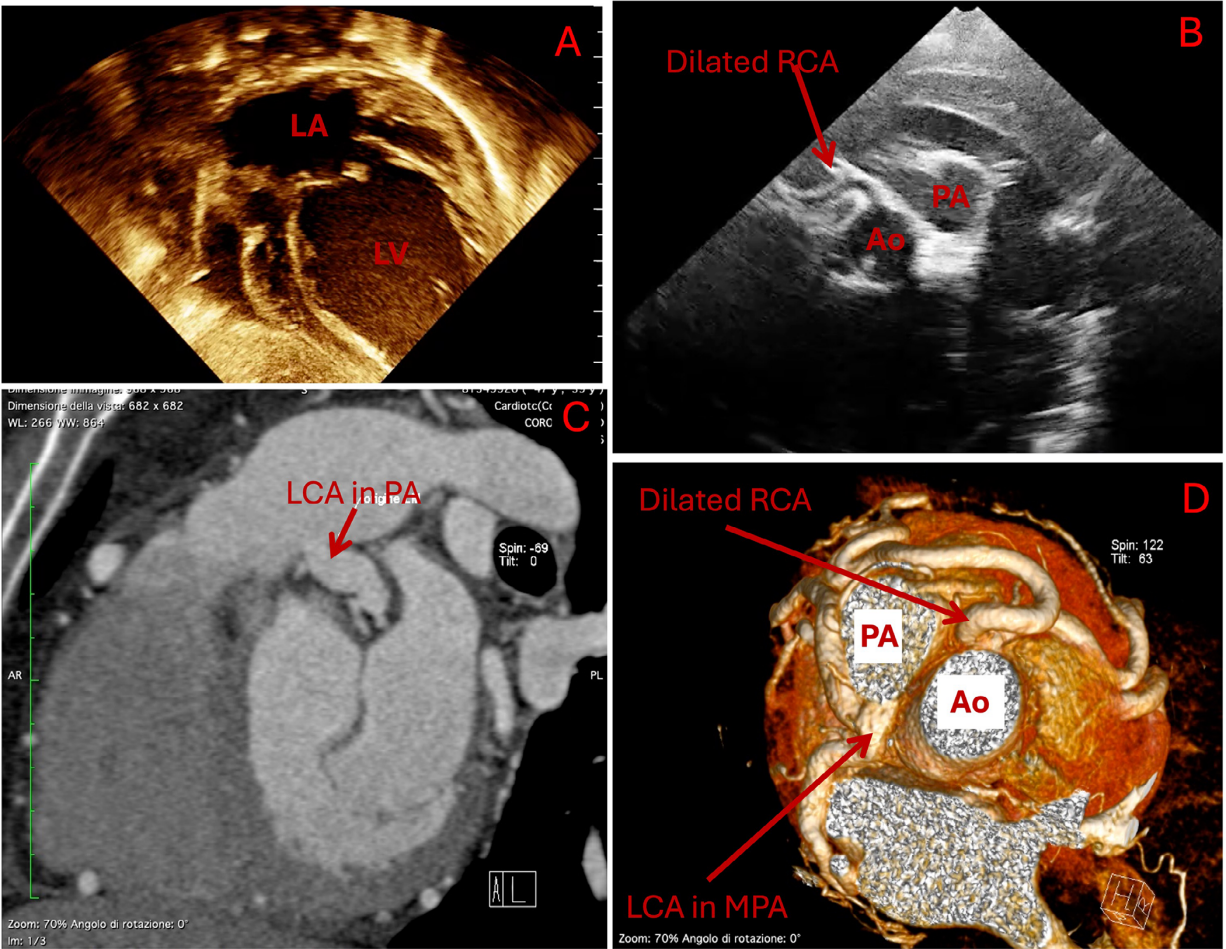
Anomalous origin of the left main coronary artery from the pulmonary artery. (A) Left ventricle was severely dilated. (B) Right coronary artery was dilated and well visible from a short‐axis view. (C) CT scan showed drainage of left coronary artery into pulmonary artery (inverted flow across the vessel). (D) 3D reconstruction of the heart. Both the coronaries were dilated. Several collateral vessels can be seen. LCA drainage in main pulmonary artery can be easily appreciated. Ao, aorta; LA, left atrium; LCA, left coronary artery; LV, left ventricle; PA, pulmonary artery; RCA, right coronary artery.

Cardiovascular multidetector CT is an excellent complementary technique for the evaluation of the origins and the entire course of coronary arteries [14]. In the last decade, the improvement of CT machines has progressively enhanced spatial resolution, reduced the time of full volume acquisition, and decreased the dose exposure. Nowadays, ECG‐gated software allows excellent resolution for studying coronary arteries and enables any post‐processing with 3D and augmented reality software (Figure 1). Thus, CT scanning is becoming a part of the imaging flow chart in the pediatric population as well as in adults [30]. Despite the emerging use of cardiac CT as advanced cardiovascular imaging, the need for ionizing radiation and iodine contrast medium still represents a major limitation for the exam repeatability. Thus, a written consent is needed to perform the exam.

If TTE can describe the coronary arteries’ origins and proximal course, cardiac CT can study in detail the lumen shape, detect the presence of intramural courses or myocardial bridges, as well as assess the relationship with the surrounding structures. By using cardiac CT, it is possible to characterize stenoses and, recently, to assess coronary flow physiology [31, 32]. This information is essential for stratifying the patient's risk and for planning surgical or interventional maneuvers [33].

**FIGURE 2:**
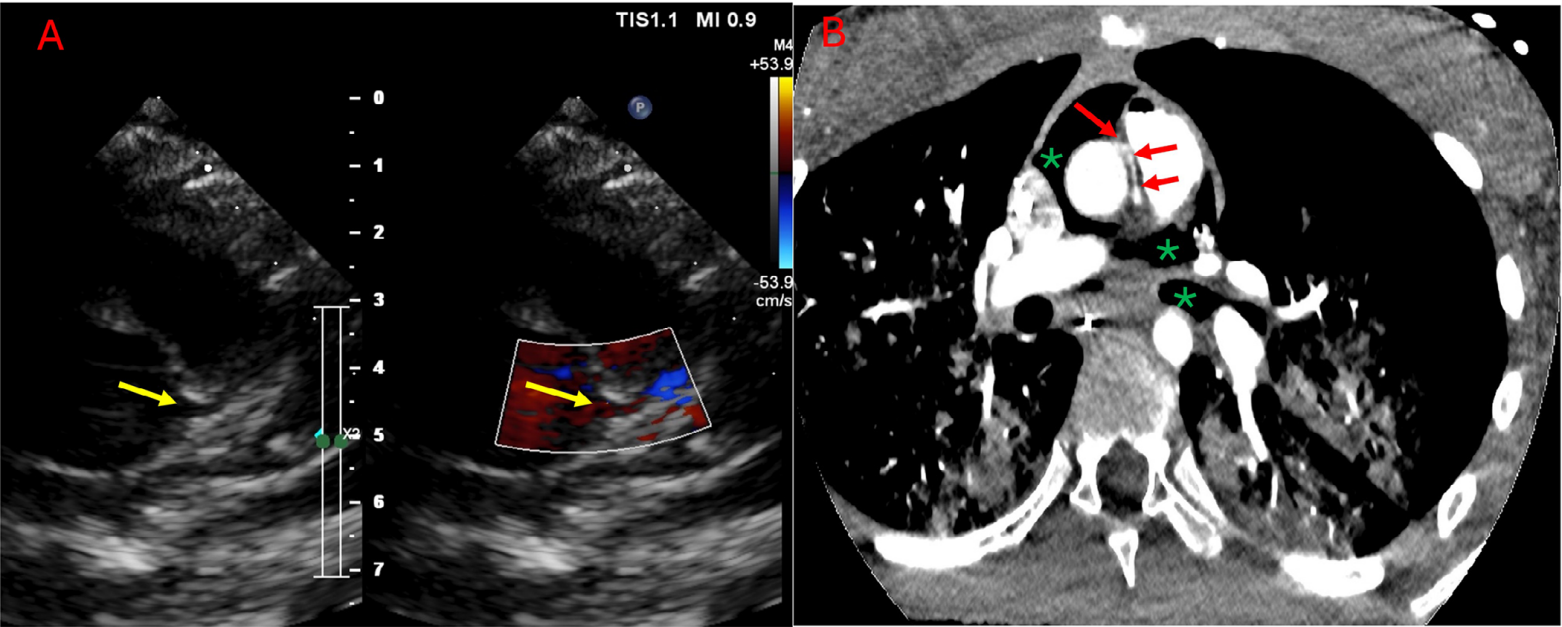
A 13‐years‐old male patient with cardiac arrest during training before a football match. Echo imagines (on the left) acquired when he was in Venoarterial—ExtraCorporeal Membrane Oxygenation seem to capture a normal origin of left main coronary artery in the left Valsalva sinus (yellow arrow). CT (on the right) diagnosed an anomalous origin of left coronary artery from opposite sinus (red arrow) with inter‐arterial course and acute take‐off. * Pneumopericardium (due to resuscitation maneuvers).

Based on the above‐described characteristics, cardiac CT has become the pivotal exam to describe the anatomical features of AAOCA: intramural course, high take‐off or slit‐like orifice, and longer intramural course (Figure 2) [34]. Based on those data, the patient can be stratified as low‐risk or high‐risk for cardiovascular events. All anatomic features of AAOCA related to myocardial ischemia can be studied: as shown in the larger multicenter study regarding AAOCA, ischemia was more common in patients with anomalous origin of the left coronary artery (AAOLCA), particularly in those who had an intramural course, a high orifice, or a slit‐like orifice; in patients with anomalous aortic origin of the right coronary artery (AAORCA) ischemia was more frequently related to longer intramural course [34].

CT can detect an intramural course with a strong correspondence with the surgical view. Miller et al. [35] compared patients with AAOCA with and without an intramural segment and found a significant difference regarding the angle of emergence (18.3 ± 3.4° vs. 48.7°) and the average height/width ratio (2.19 vs. 1.03).

To study the coronary orifice origin, the eccentricity index (minor diameter/major diameter ratio) is useful for defining an oval or slit‐like ostial shape, which are major determinant of ischemia in patients with AAOCA [36]. Once an en‐face view of the ostium is obtained, it is possible to determine the ratio between minor diameter a major diameter ratio and to calculate the eccentricity index. A value < 0.5 is usually associated with high‐risk anatomy. Interestingly, in pediatric patients, the eccentricity index can get worse during the growth. Thus, serial evaluations should be considered in pre‐adolescent subjects [36]. On the other hand, this might be one of the reasons for a low incidence of sudden cardiac death in prepubertal subjects [36].

The length of the intramural course was significantly associated with a higher risk of myocardial ischemia [37]. Molossi et al. [37] found that the presence of an intramural length course of 3.9 mm or higher in AAOCA could predict the likelihood that a lesion would be considered “high‐risk” with reasonable sensitivity (77%) and specificity (75%).

**FIGURE 3:**
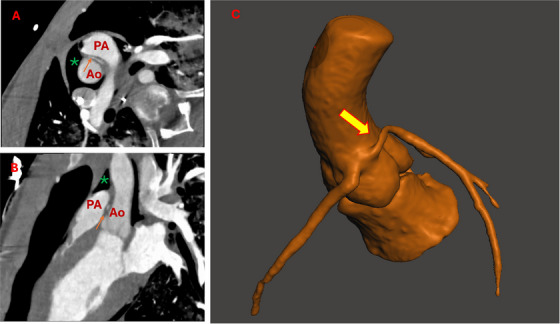
The same patient shown in Figure 2. (A) After a short, slit‐like and intramural course, left coronary artery showed a long interarterial course (arrow). (B) In that tract, the vessel was significantly eccentric (arrow). (C) 3D reconstruction of aorta and left coronary artery. The elliptic shape of left coronary artery along the right coronary sinus can be easily seen (interarterial course, arrow). * Pneumopericardium; Ao, aorta; PA, pulmonary artery.

When available, advanced CT‐post processing imaging software permits virtual endoluminal reconstruction of the aortic root [38]. Thanks to these precise 3D images, clinicians can define the length and course of intramural segments, and surgeons can simulate surgical maneuvers by determining the length of unroofing needed, potential interferences with intracoronary commissure, and the best point for creating neo‐ostia distal to the intramural segments [38] (Figure 3). All intramural segments detected by these endoluminal views present a curvilinear band of the intramural coronary artery segment, that corresponds surgically to an intimal thickening of the aortic root forming the roof of the intramural segment [38, 39].

## 4 Cardiac Magnetic Resonance

CMR can be considered a complementary imaging method to echocardiography and cardiac CT for the evaluation of AAOCA. Compared to echo, it offers a better spatial resolution that allows precise visualization of the origin and the entire course of coronary arteries [2, 14]. CMR shows coronary anatomy in a standard axial, sagittal, and coronal projection, or, with post‐processing algorithms, creates “echo equivalent” images for comparison [14]. Compared to CT, CMR provides additional relevant morphologic features, like ventricular function, valvular function, regional myocardial contractility and viability, presence of scars and blood flow data. These data play a pivotal role in the decisional workflow as well as in the follow‐up of AAOCA patients [40]. Interestingly, several anatomical and functional data can be obtained without use of contrast agents. Thus, CMR can be considered as a second‐line imaging tool in children, to avoid use of contrast medium and ionizing radiations [40, 41].

On the other hand, gadolinium‐based agents enhance the quality of the images and help identify areas of fibrosis. This finding may represent either a marker of ischemia, or a focus for malignant arrhythmias [4, 42].

Currently, CMR can also be used for stress imaging. Compared to nuclear perfusion imaging, it overcomes several limitations of that technique, such as exposition to ionizing radiation, low spatial resolution, attenuation artifacts related to the body wall and diaphragm, and a relatively high incidence of false positive results. Thus, together with stress echo, stress CMR might be the preferred non‐invasive method of choice regarding the diagnosis of myocardial ischemia in AAOCA [43, 44]. Although physical exercise is preferable, a pharmacologic test might be needed to overcome patient‐based or technical limitations. Several protocols are available in the literature. However, dynamic obstruction at the ostium or at the proximal course of the coronary artery are the most common triggers for stress‐induced ischemia [43]. Thus, dobutamine seems to better replicate this pathophysiological pathway, by increasing contractility and reducing systemic vascular resistance, similar to what occurs during physical exercise [43, 44, 45]. Doan et al. [46] performed 224 dobutamine cardiac stress CMR on 182 pediatric AAOCA patients at a median age of 14 years. Feasibility was 98.9%, and sedation was used in 17.5% of the exams. No major adverse events were reported, and the minor complication rate was 12.5% (nausea, vomiting, hypertension, anxiety, skin rash) [46]. Inducible myocardial hypoperfusion was detected in 31 (14%) patients, of which wall motion abnormalities were evident in 13 (42%), and late gadolinium enhancement was found in 3 subjects [46]. These patients underwent surgical treatment of AAOCA, and no symptoms were reported during follow‐up in those with negative test [46]. Some studies have evaluated the role of feature tracking in exercise CMR. Despite the role of speckle tracking by echo is well established, no study clearly demonstrated the role of feature tracking in pediatric AAOCA patients. In a more recent retrospective study, the feasibility of the dobutamine stress CMR was confirmed in 54 patients with AAORCA, 13 of them with high‐risk anatomies. However, 18 of them reported exercise‐induced symptoms [47, 48]. Probably, exercise‐induced tachycardia and a lower frame rate by CMR compared to echo might limit the accuracy of feature tracking in this setting.

In conclusion, exercise CMR is a valuable tool to assess inducible ischemia in AAOCA patients. The positive predictive value of this tool is high. Agrawal et al. [49] compared FFR with exercise CMR data. Interestingly, all positive CMR tests reported significant reduction in FFR after dobutamine infusion. Thus, CMR can significantly contribute to risk stratification and decision‐making in children with complex coronary anomalies [49]. In the case of a positive test, surgery should be strongly considered. Patients with negative tests might continue with a non‐invasive follow‐up and serial stress CMR assessments can be safely considered. In corrected AAOCA patients, CMR can be effectively used as well [50]. CMR and stress CMR require adequate equipment, skilled operators, and dedicated tools [43]. Therefore, their availability for a large‐scale use is low, and patients should be referred to dedicated and high‐volume centers.

## 5 Role of the Angiography (FFR/iFR and IVUS)

**FIGURE 4:**
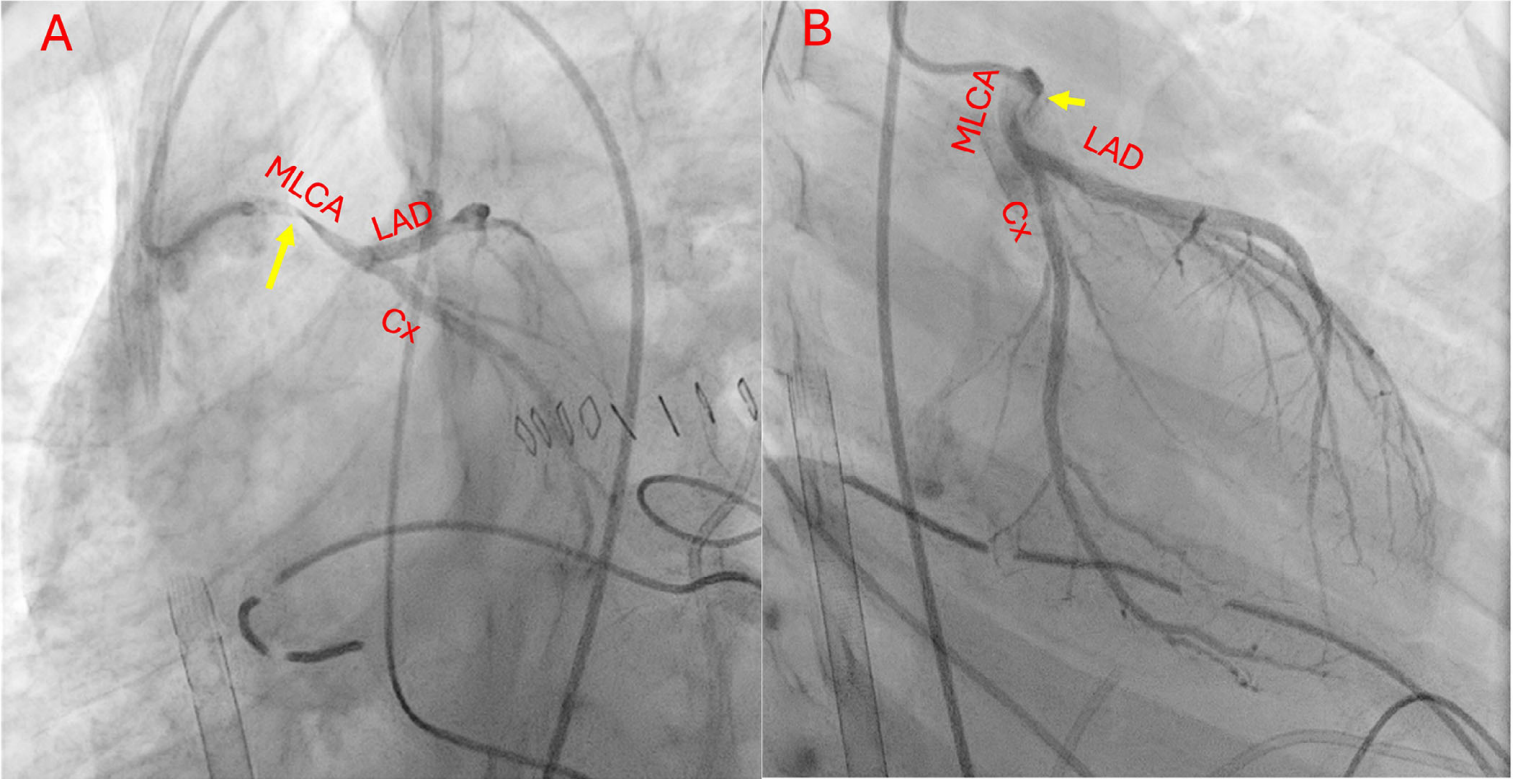
Angiography confirmed the anomalous origin of left coronary artery. (A) Left anterior caudal view, (B) right anterior caudal view. The MLCA showed an intramural course (corresponding to a thinned calibre of the first part of the left coronary artery, yellow arrow). Normal arborization of the peripheral branches of the left coronary artery was also found. Cx, circumflex artery, LCA, left coronary artery; MLCA, main left coronary artery.

Cardiac angiography has been considered for decades the method of choice for evaluating coronary anatomy. Fortunately, improvements in non‐invasive techniques have reduced the need for coronary angiography, as CT and CMR have proven to be more useful for describing spatial relationship with surrounding structures and for creating 3D models and holographic views of coronary arteries [51]. Nowadays, coronary angiography is reserved for interventional procedures or when non‐invasive assessment is inconclusive [51]. In the latter case, functional invasive tools are often needed to achieve a detailed diagnosis (Figure 4). The most commonly used tools in AAOCA patients are intravascular ultrasound imaging (IVUS), fractional flow reserve (FFR), and instantaneous wave‐free ratio (iFR) [29].

In adult patients, IVUS has been considered the gold standard for the evaluation of coronary anomalies [52, 53]. Conventional IVUS probes could provide images that can be processed online or offline to calculate effective coronary lumen area, eccentricity index, and to detect intramural or intramuscular courses. This information may be very helpful for AAOCA patients.

Angelini et al. [52] used IVUS to confirm intramural course, the lesion's length, and the changes in coronary lumen during the cardiac cycles in AAORCA patients. Additionally, they studied the changes in coronary lumen during pharmacologic stress tests (by using adenosine or dobutamine), demonstrating cross‐sectional area variations in simulated exercise settings [52]. In this study, 42/67 patients were treated by stenting of the intramural course. The rate of re‐stenosis in the follow‐up was 10% [52].

In pediatric patients, IVUS use was reported in small case series and different clinical settings [54, 55]. Few data are available for AAOCA patients as well. Agrawal et al. [56] reported data from eight pediatric AAOCA patients. They concluded that the association with IVUS and FFR can be safely performed in pediatric patients (mean age was 13.1 years, range 2.6–18.7 years) and provide additional and essential information for decision‐making process [56]. Since IVUS system could be advanced in a 5 Fr guiding catheter, there is little incidence of complications in even smaller patients, depending on their femoral and coronary arterial sizes [56].

**FIGURE 5:**
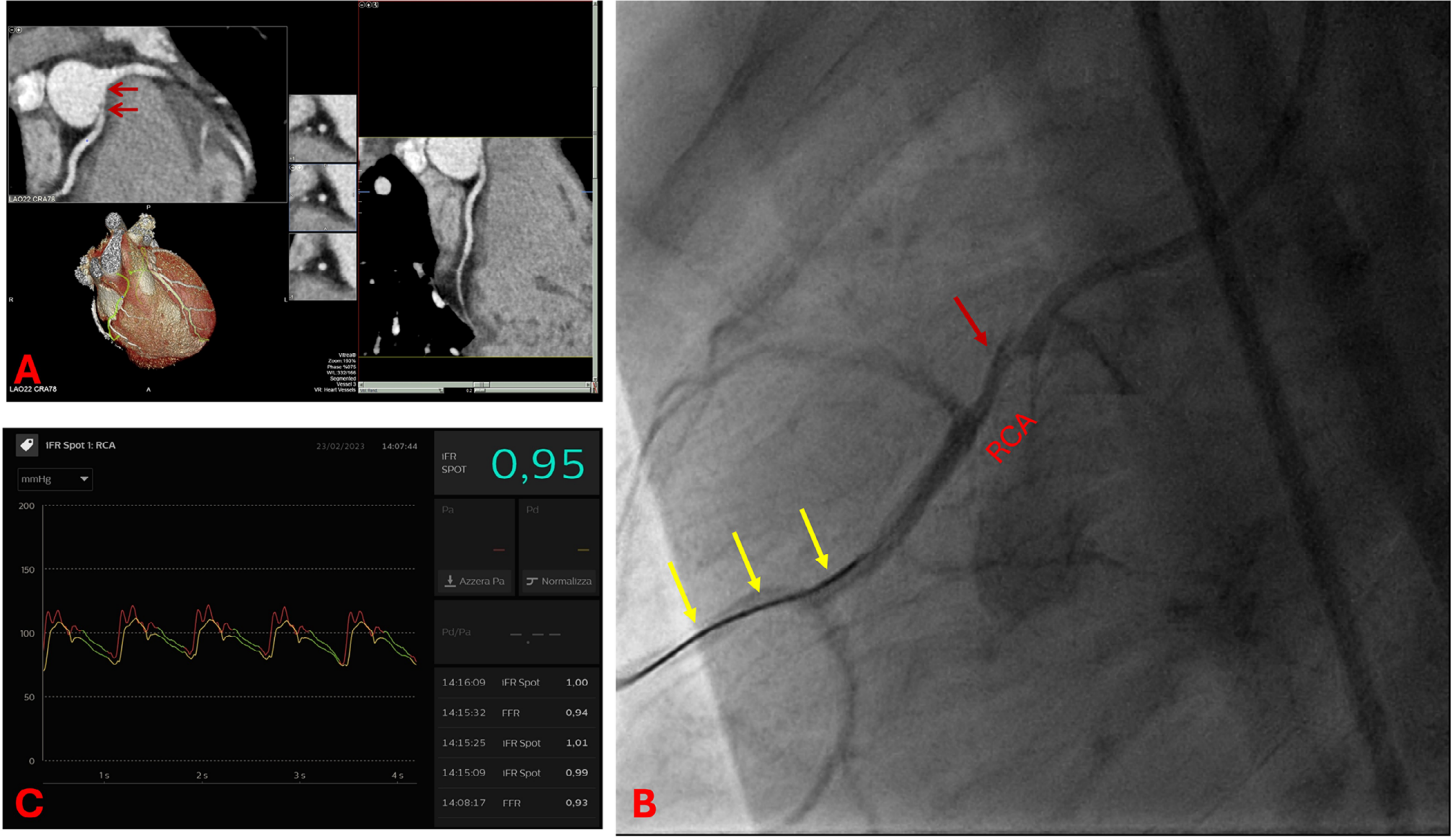
Anomalous origin of right coronary artery (RCA) from the left aortic sinus. (A) CT scan showed a long intramural course behind the right ventricular infundibulum. (B) Selective right coronary angiography showed an irregular shape of the proximal tract (red arrow). The tip of pressure guidewire (yellow arrows) was distal in the vessel. However, iFR plot (C) showed values within normal limits (> 0.93).

FFR is a pressure‐derived index of physiological significance of a coronary stenosis. It represents the ratio bteween mean coronary artery pressure (measured by a coronary artery guidewire distal to a stenosis) and mean aortic pressure (measured simultaneously by the guiding catheter in the aortic root) [57]. This parameter is routinely used in borderline coronary lesions, to guide percutaneous coronary interventions in coronary artery diseases [58]. Normally, FFR value should be ideally 1.0 (no difference between proximal and distal pressure). Current evidence indicates 0.80 as cut‐off value for significant stenotic lesion [58]. Despite this value was defined on atheromatic plaques settings, the same value was applied to AAOCA patients without a case‐specific validation [59, 60]. FFR can be used during stress test, too. In an atheromatic lesions, adenosine can be safely and effectively used to induce diffuse coronary vasodilatation, unmasking exercise‐induced stenoses. In AAOCA patients the pathophysiology is different, being the lesion ostial or very proximal and often dependent by ab‐extrinsic compressions (intramural or interarterial courses) [61]. Thus, dobutamine should be preferred in these patients [56]. Recently, iFR was proposed as alternative method to quantify coronary stenoses. It can be calculated with the same tools used for FFR, thus can be both achieved during the same test. iFR is calculated as the ratio of blood pressure distal and proximal to a coronary artery stenosis during the diastolic phase. Differently from FFR, iFR studies a specific phase of coronary flow phase, the wave‐free period. In this period, starting at the 25% of diastolic phase and ending 5 ms before systole, microvascular resistances result steadily low. Therefore, this parameter might be more indicative of the effective myocardial perfusion. Normal values for iFR are ratio between 0.93 and 1.0. The test is positive for values < 0.86. Results between 0.93 and 0.86 are considered in “grey zone” [56, 62]. Few data are available on iFR in AAOCA [63, 64, 65, 66] (Figure 5). Methods and cut‐off were the same used in adults with plaque‐related ischemia. However, dedicated study should be built to validate these data in AAOCA setting. The assessment of invasive coronary physiology by FFR/iFR can be considered in patients with conflicting data between imaging and symptoms [67]. In a populatiosn of eight patients (three AAOLCA and five AAORCA) iFR and FFR were suggestive of a significantly impaired coronary flow in four of them, and a surgical repair were requested [67]. The repetition of these exams after surgery revealed the normalization of these indexes [67]. On the other hand, a functional assessment can be considered to grant sport activity in AAOCA patient without high‐risk anatomic features.

In children with AAOCA, the use of invasive studies is not routinely performed because of the need of deep sedation, potential challenges in ostia cannulation or iatrogenic injuries on coronaries or femoral vessels. Thus, Jiang and colleagues [65] introduced a novel noninvasive option using a CT derived method called fluid‐structure interaction (FSI) and compared it to iFR measured invasively. In six adult patients with AAORCA, FSI models accurately simulated the iFR at rest and after dobutamine stress using only blood pressure, heart rate and noninvasive imaging data [65]. Although this study presents several limitations, mostly regarding the “simplification and smoothing” of the model, FSI deserves future further investigations on this topic.

## 6 Guidelines: Comparative Assessment

The diagnosis of coronary anomaly raises several questions regarding the diagnostic process and the most appropriate therapeutic strategy for the patient.

Regarding which patient should be recommended/considered for cardiac surgery is matter of debate. In the latest ESC guidelines (Table 1), a class I recommendation for surgery is limited to the presence of angina associated AND inducible ischemia in the corresponding AAOCA territory, OR in the presence of anatomical high‐risk features (class I) [12]. In the other cases, decision depends on the following variables:

- the coronary involved (AAOLCA vs. AAORCA),
- the presence of specific anatomical risk factors (acute take‐off, high take‐off, slit‐like orifice),
- symptoms or inducible ischemia,
- young age (< 35 years).

**TABLE 1:** Last ESC guidelines [12] regarding AAOCA surgical indications.

	AAOLCA	AAORCA
Symptoms + Ischemia or Anatomy* +	YES (I)	YES (I)
Symptoms − Ischemia +	YES (IIa)	YES (IIa)
Symptoms + Ischemia −	YES (IIb)	YES (IIb)
Symptoms − Ischemia − Anatomy* +	YES (IIb)	/
Young Age	YES (IIb)	NO (III)

*Note*: * High risk anatomies: intramural course, slit‐like ostium, acute take‐off, high take‐off >1 cm over ST junction.

The latest American Heart Academy (AHA) guidelines suggest different factor compared to ESC guidelines regarding the decision‐making process: they share the necessity for intervention in the presence of symptoms and inducible ischemia and identify AAOLCA as the highest risk category. However, differently from the previous guidelines, they introduce ventricular arrhythmia as an indication for surgery (IIa). Furthermore, they do not exclude cardiac surgery for AAORCA, even in the absence of anatomical risk factors (IIb) [68].

Interestingly, symptoms represent a weak indication for cardiac surgery in low risk anatomies. In fact, chest pain is frequent and nonspecific in the paediatric population, often related to musculoskeletal issues. In general population, only in 0.3%–8% of young patients a cardiac etiology was identified [69]. Similarly, neurogenic syncope is largely prevalent in paediatrics (95% of the patients). On the other hand, only 50% of patients with AAOCA are symptomatic, and a significant percentage of patients may present with sudden cardiac death as the first symptom [71, 72]. Generally, AAOLCAs tend to be more symptomatic than AAORCAs, which, although typically considered more benign anomalies, are more often asymptomatic until a sudden event occurs [34].

Although cardiac surgery is generally safe and effective in expert hands, it may not fully resolve the myocardial ischemia, with revision surgery required in 2%–10% of cases [34, 73, 74, 75, 76]. Furthermore, a iatrogenic aortic regurgitation might complicate surgical manoeuvres, in particular in unroofing procedures [76], and sudden deaths during sports activity in AAOCA patients despite surgical correction were reported [77]. Finally, a current challenge is how to manage sports activity in AAOCA. AHA guidelines consider eligible for sport activity only AAORCA patients without symptoms and inducible ischemia (IIa); and surgically corrected AAOCA >3 months after surgery, after a negative exercise test (class IIb) [78].

In contrast, the 2020 ESC guidelines for sport activity make no distinction between AAORCA and AAOLCA, sports eligibility may be reserved only for those who are asymptomatic, without anatomical risk factors, and without inducible ischemia (IIb), or for those who, as per the previous guidelines, underwent cardiac surgery at least 3 months prior, are asymptomatic, and do not show inducible ischemia or ventricular arrhythmias during a maximal stress test (IIb) [79].

Although intense physical activity is the condition that most exposes a patient with congenital coronary anomaly to risk, physical activity restriction does not necessarily protect the patient from sudden events, which may also occur at rest or during mild physical activity. Additionally, it is important to underline the emotional and psychological consequences of depriving the patient of sports, as well as the well‐known effects on physical health [2, 80].

**FIGURE 6:**
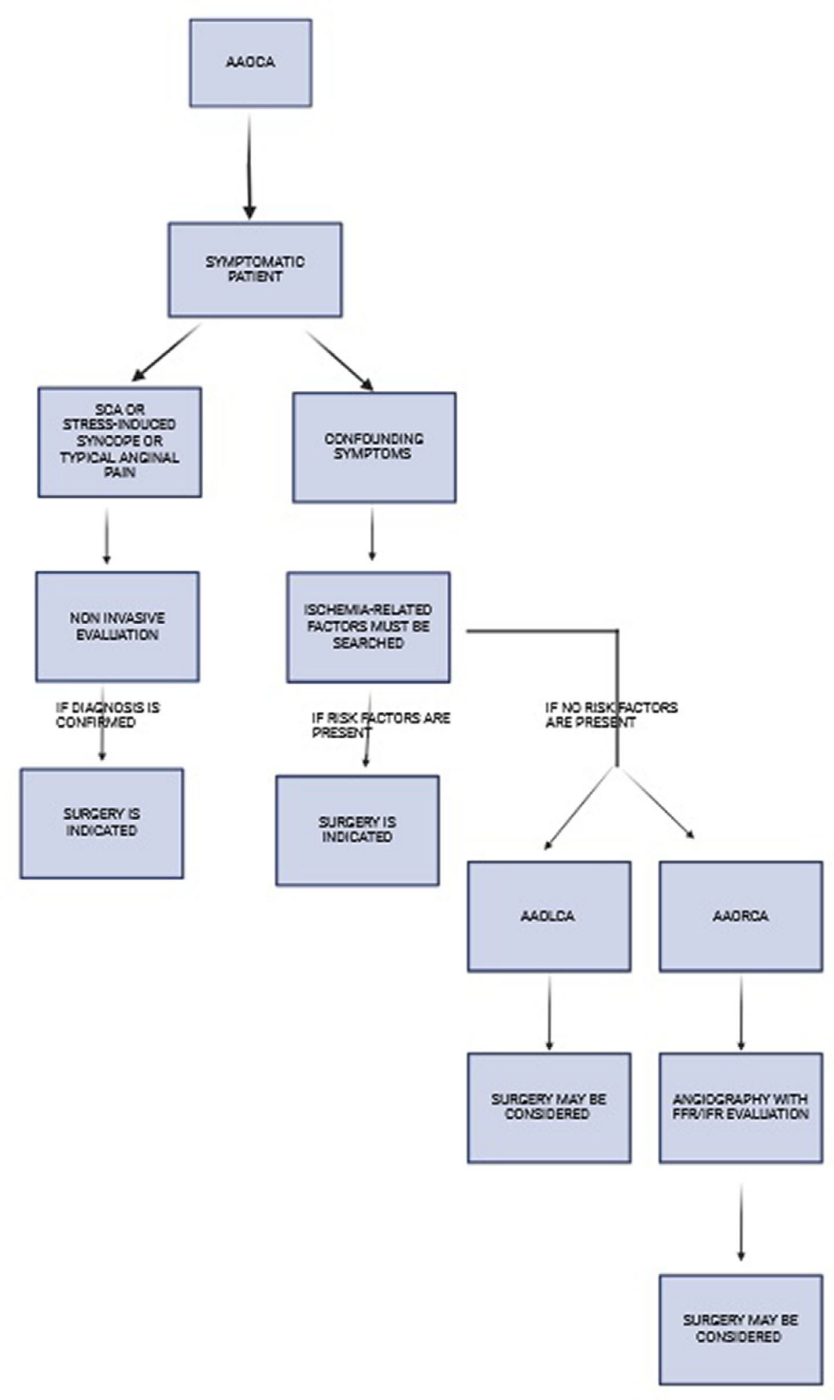
Diagnostic management in patients with AAOCA with symptoms.

**FIGURE 7:**
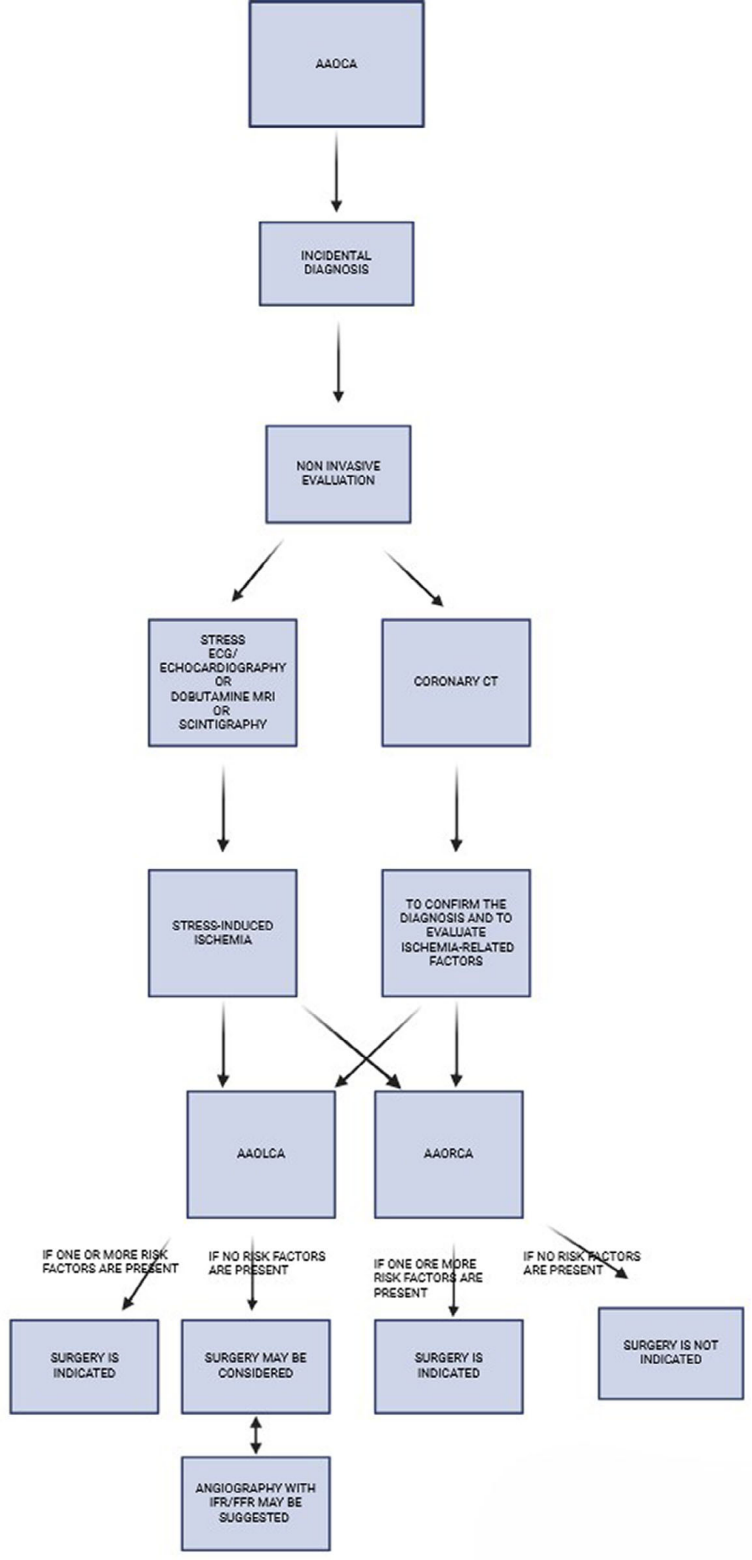
Diagnostic management in patients with AAOCA without symptoms. SCA, sudden cardiac arrest.

To summarize, we report a possible management flow‐chart regarding management of AAOCA patients (Figures 6 and 7).

Probably, a more effective risk stratification might further improve the ability to stratify patients with low risk for sudden events. Future studies should explore the possible role of iFR (CT‐ or angio‐based), advanced stress imaging (stress‐CMR and echo‐stress) to effectively screen these patients.

## 7 Conclusion

AAOCA represents a real challenge to diagnostic workflow and treatment. Despite often the diagnosis might be incidental and most patients remain asymptomatic, AAOCA still remains a major cause of sudden cardiac death in young and “healthy” people, often as first symptom. Recent dedicated international guidelines helped to standardize the approach to AAOCA. However, experience and clinical evidence on pediatric population are scarce. Echocardiography remains the first line diagnostic procedure, but CT and MRI are mandatory to confirm the diagnosis, to stratify the risk, and to plan surgery. Assessing invasive coronary physiology by FFR and iFR might provide additional information in complex cases or in patients with a mismatch between anatomy and symptoms.

## Conflicts of Interest

The authors declare no conflicts of interest.

## Data Availability

Data sharing is not applicable to this article as no datasets were generated or analyzed during the current study.
